# Supplementary material for: Investigating the role of perceived autonomy support in moderating the association between diabetes stigma and psychological and diabetes self-management outcomes among adults with type 2 diabetes in Ghana
Source: PLOS Glob Public Health. 2026 Feb 19;6(2):e0006027. doi: 10.1371/journal.pgph.0006027 (PMC12919806; doi:10.1371/journal.pgph.0006027)
Supplement: Attachment — Submitted filename: Review.docx [file pgph.0006027.s003.docx]

## General Comments

1) Please include line numbers in the manuscript, as this greatly facilitates precise and accurate feedback.
2) The concept of “diabetes distress” is used but never defined. A brief definition would help contextualize the construct for readers.

3) On page 3, the statement *“In Ghana, diabetes-related stigma may manifest through being perceived as HIV-positive”* may inadvertently reinforce stigma associated with HIV by implying that such an association is inherently negative. This issue might have been clearer if more contextual information about Ghanaian cultural beliefs had been provided.
   Additionally, further explanation of the role of autonomy in Ghanaian culture and its collectivist society would help readers understand why autonomy support from healthcare professionals received relatively limited attention.
   More broadly, stigma is closely linked to societal stereotypes and culturally shared assumptions. Since this point was only briefly mentioned at the end of the introduction and not developed in the discussion, expanding on it could significantly strengthen the manuscript.
4) In the sociodemographic variables, only gender appears to be reported. Should **sex** also be included?
5) The quality of the figures (particularly Figures 1 and 2) could be improved to enhance readability.

## Abstract

1) In the first sentence, adding concrete examples of factors that mitigate negative impacts of stigma would improve clarity.
2) It is generally recommended not to use abbreviations in the abstract.
3) The psychological outcomes examined (depression, anxiety, diabetes distress) are not introduced in the abstract’s introduction, which limits contextual understanding.

## Specific Comments

1) The following statements require references (page 3):

- “These lifestyle changes following diabetes diagnosis can be physically and psychosocially daunting…”
- “This is striking given the critical role healthcare professionals play in educating, supporting, and empowering adults with T2D.”

2) When concepts are introduced, providing examples would improve clarity (e.g., “lifestyle changes” (15,16) page 4; “healthy behaviors” page 4).
3) The citation **(Ryan & Deci, 2000)** on page 4 is not formatted consistently with the other references.
4) A closing parenthesis is missing in the eligibility criteria section after *“(the most popular dialect in Ghana (21, 22))”* on page 5.
5) A colon **":"** is missing after the heading *“Perception of autonomy support”* on page 7.
6) In Table 1 (pages 9–10), the “monthly income” variable totals **188 participants**, not 190.
7) In Table 3 (page 13), the variable labels are inconsistently capitalized:

- Models 1 and 4 begin with lowercase.
- Models 2 and 3 begin with uppercase.
  Harmonization is recommended.

8) Adding **notes below Table 3** to define abbreviations would help readers.
9) The following references should be harmonized to match the formatting of the others: **1, 2, 4, 29, 37, 40, 48**.
