# Supplementary material for: Investigating the role of perceived autonomy support in moderating the association between diabetes stigma and psychological and diabetes self-management outcomes among adults with type 2 diabetes in Ghana
Source: PLOS Glob Public Health. 2026 Feb 19;6(2):e0006027. doi: 10.1371/journal.pgph.0006027 (PMC12919806; doi:10.1371/journal.pgph.0006027)
Supplement: Attachment — Submitted filename: response to reviewers comments.docx [file pgph.0006027.s005.docx]

Dear Editor,

Thank you for the opportunity to revise our manuscript entitled “Investigating the role of perceived autonomy support in moderating the association between diabetes stigma and psychological and diabetes self-management outcomes among adults with type 2 diabetes in Ghana.” which we submitted to the *PLOS Global Public Health*. We appreciate the time and effort that the editor and reviewers have taken to evaluate our work and provide constructive feedback. We have carefully considered the insightful comments made by the reviewers and have made corresponding amendments to reflect the suggestions made. Changes to the manuscript body are highlighted in yellow and our responses to the reviewers’ comments are summarized below.

**Reviewer #1**

**Comment 1**: Please include line numbers in the manuscript, as this greatly facilitates precise and accurate feedback.

**Response**: Thank you. We have now added line numbers to the manuscript.

**Comment 2**: The concept of “diabetes distress” is used but never defined. A brief definition would help contextualize the construct for readers.

**Response**: We have now included a standard definition of diabetes distress to enhance clarity.

**Comment 3**: On page 3, the statement “In Ghana, diabetes-related stigma may manifest through being perceived as HIV-positive” may inadvertently reinforce stigma associated with HIV by implying that such an association is inherently negative. This issue might have been clearer if more contextual information about Ghanaian cultural beliefs had been provided.

**Response**: We agree with the reviewer and appreciate their comment. We have now modified the statement to enhance clarity. “In Ghana, diabetes-related stigma may emerge from illness interpretations in which visible symptoms of diabetes, such as sudden weight loss in the early stages of diagnosis and delayed wound healing at later stages, are socially interpreted through moralized or spiritual lens, leading to misidentification with other highly stigmatized conditions (e.g., HIV), accusations of witchcraft, and, consequently, social ostracism”

**Comment 4**: Additionally, further explanation of the role of autonomy in Ghanaian culture and its collectivist society would help readers understand why autonomy support from healthcare professionals received relatively limited attention. More broadly, stigma is closely linked to societal stereotypes and culturally shared assumptions. Since this point was only briefly mentioned at the end of the introduction and not developed in the discussion, expanding on it could significantly strengthen the manuscript.

**Response**: We have now added two sentences towards the end of the intro to shed more light on the nature of collectivist society and the concept of autonomy in this society.

**Comment 5**: In the sociodemographic variables, only gender appears to be reported. Should sex also be included?

**Response**: We thank the reviewer for this comment. Unfortunately, we only collected data on gender and not sex assigned at birth. Gender as a social construct was considered more pertinent to the psychosocial construct we investigated.

**Comment 6**: The quality of the figures (particularly Figures 1 and 2) could be improved to enhance readability.

**Response**: We have now included high quality figures.

**Comment 7**: In the first sentence (of the abstract), adding concrete examples of factors that mitigate negative impacts of stigma would improve clarity.

**Response**: We have now listed two concrete examples of factors that mitigate the impact of stigma.

**Comment 8**: It is generally recommended not to use abbreviations in the abstract.

**Response**: We have now written all abbreviations in full at first use.

**Comment 9**: The psychological outcomes examined (depression, anxiety, diabetes distress) are not introduced in the abstract’s introduction, which limits contextual understanding.

**Response**: We have now clearly stated what the psychological outcomes are

**Comment 10**: The following statements require references (page 3): “These lifestyle changes following diabetes diagnosis can be physically and psychosocially daunting…” and “This is striking given the critical role healthcare professionals play in educating, supporting, and empowering adults with T2D.”

**Response**: We have now included the relevant citations.

**Comment 11**: When concepts are introduced, providing examples would improve clarity (e.g., “lifestyle changes” (15,16) page 4; “healthy behaviors” page 4).

**Response**: We have clarified what we mean by these concepts

**Comment 12**: The citation (Ryan & Deci, 2000) on page 4 is not formatted consistently with the other references.

**Response**: Thank you. This has been corrected.

**Comment 13**: A closing parenthesis is missing in the eligibility criteria section after “(the most popular dialect in Ghana (21, 22))” on page 5.

**Response**: Thank you. This has now been corrected.

**Comment 14**: A colon ":" is missing after the heading “Perception of autonomy support” on page 7.

**Response**: Thank you. This has now been corrected.

**Comment 15**: In Table 1 (pages 9–10), the “monthly income” variable totals 188 participants, not 190.

**Response**: We had two missing data for that variable. This has now been included in the table.

**Comment 16**: In Table 3 (page 13), the variable labels are inconsistently capitalized: Models 1 and 4 begin with lowercase. Models 2 and 3 begin with uppercase. Harmonization is recommended.

**Response**: We have now corrected this inconsistency.

**Comment 17**: Adding notes below Table 3 to define abbreviations would help readers.

**Response**: Notes have now been added to table 3.

**Comment 18**: The following references should be harmonized to match the formatting of the others: 1, 2, 4, 29, 37, 40, 48.

**Response**: We have reviewed and corrected all references for consistency. Reference #1 is a webpage so would have a different format from the others.

**Reviewer #2**

**Comment 1**: Emphasize that this is one of the first studies to explore autonomy support in the context of diabetes stigma in Ghana, a collectivist society (If so). This would underscore the study's unique contribution.

**Response**: The novelty of the study is now emphasized in the first two sentences of the discussion section.

**Comment 2**: The manuscript briefly mentions the hierarchical and collectivist nature of Ghanaian society. Expanding on how this cultural context might influence autonomy support and diabetes stigma could provide a richer understanding of the research.

**Response**: We have now highlighted that the cultural context may influence people to not value autonomy in the same way as individualistic societies might. This has been highlighted in the intro and discussion sections on pages 4 and 16.

**Comment 3**: “Whereas autonomy support is universal, it may not present in the same manner and may not be as beneficial across cultures (19)”. While the authors mention ross cultures, there should have been many references from different cultures.

**Response**: The cited reference is the source of the idea presented in the statement and not necessarily referencing specific cultures. For clarity, we have now included another reference as suggested.

**Comment 4**: While the manuscript mentions purposive and convenience sampling, it could provide more details about how participants were selected and whether this approach might introduce bias. For example, were there any efforts to ensure diversity in the sample (e.g., age, gender, socioeconomic status)? This has to be stated in the limitation section.

**Response**: We have amended the method section to provide more details about the sampling procedure. We have also noted the use of non-probability sampling as potential source of bias in the limitation section.

**Comment 5**: Meanwhile, I suggest the authors interpret the socioeconomic characteristics of the study population in light of those of studies from other parts of the world with diverse cultures (particularly from high-income countries). This should have been added in the discussion section by using the following suggested articles.

https://doi.org/10.1155/2020/9408316

https://doi.org/10.1016/j.clinthera.2016.07.006

https://doi.org/10.2337/ds23-0013

**Response**: We appreciate the reviewer’s suggestion to discuss socio-economic characteristics of the study population and for providing useful articles for reference. However, we believe that discussing the socioeconomic characteristics of the study sample will distract from the core aims of the manuscript (i.e., perceived autonomy support and stigma). We have another paper under review elsewhere where we do a deep dive on this topic.

**Comment 6**: The manuscript states that surveys were administered via the Qualtrics mobile app. It would be helpful to clarify whether the app was used for both researcher-administered and self-administered surveys, and how data quality was ensured during these processes.

**Response**: We have now clarified that while we offered participants the option to self-administer the survey, none chose this option. So Qualtrics was only used for researcher-administered questionnaire.

**Comment 7**: The statistical methods are well-described, but the rationale for choosing hierarchical multivariable linear regression and the Johnson-Neyman technique could be briefly explained for readers unfamiliar with these methods.

**Response**: We have provided additional rationale to justify the choice of our analytical techniques.

**Comment 8**: While ethical approval and informed consent are mentioned, the manuscript could elaborate on how participant confidentiality and data security were maintained during the study.

**Response**: We have added more information on the steps we took to ensure confidentiality and data security under the section “ethical approval”.

**Comment 9**: “Table 1 shows descriptive statistics.” This should be Table 2.

**Response**: Thank you. We have corrected this error.

**Comment 10**: Table 2: “Perceived autonomy support was negatively and significantly associated with anxiety (r=-0.23) and depressive symptoms (r=-0.27). Participants who reported greater perceived autonomy support tend to engage in better diabetes self-management (r=0.24).” The correlation r<0.3 is usually considered weak. The authors should state this in the text as well.

**Response**: We have rephrased the statements to highlight that the correlation was weak.

**Comment 11**: Table 3: p-value of DSAS-2 in block 2 written as <0.00001, and this should be corrected as <0.0001. Please check that all the decimals are consistent.

**Response**: Thank you. We have corrected this error.

**Comment 12**: Figures: Ensure figures are high-resolution for better readability, especially for publication. Use consistent font sizes, styles, and colors across all figures for a professional appearance. The captions should provide a brief explanation of the figure's content, including the key findings and any critical annotations.

**Response**: We have now included higher resolution figures in the manuscript and have added more details to the captions

**Comment 13**: Figure 1

Clarity of Labels: The axis labels are not fully descriptive. For example, "Diabetes-related stigma" could be labeled as "Diabetes Stigma (DSAS-2 Score)" for clarity. Similarly, "Anxiety symptoms" and "Depressive symptoms" could be labeled with their respective scales (e.g., "Anxiety Symptoms (PROMIS Anxiety T-Score)" and "Depressive Symptoms (PROMIS Depression T-Score)").

Data Points: The scatter plot appears cluttered, making it difficult to interpret the relationships. Consider reducing the size of the data points or using transparency to make overlapping points more distinguishable.

Trend Lines: The trend lines are present, but their labels or legends are missing. Adding a legend or annotations to clarify the meaning of the trend lines would improve readability.

**Response**: We appreciate this detailed feedback. We have amended figure 1 to reflect your suggestions. We have clarified the meaning of the trend lines in the figure’s caption.

**Comment 14**: Figure 2

Axis Labels: The x-axis label "T2D stigma z-scores" could be expanded to "Diabetes Stigma (Standardized Z-Scores)" for clarity. The y-axis label "Autonomy support" is unclear. It could be updated to "Interaction Effect with Diabetes Stigma" to reflect better the data being presented.

Legend: The figure lacks a legend explaining the meaning of the different lines (e.g., "Mean z-score," "1 SD," etc.). Adding a legend would make the figure more straightforward to interpret.

Visual Design: The figure could benefit from a more apparent distinction between the shaded areas (e.g., using different colors or patterns for "n.s." and "p < .05"). The dashed vertical line could be labeled to indicate its significance (e.g., "Threshold for significant moderation effect").

**Response**: We thank the reviewer for these useful observations. Please note that figure 2 is a standard “spotlight” interaction plot. We have added additional details in the caption to clarify the plot’s interpretation. We have also modified the axis labels.

**Comment 15**: Figure 3

Axis Labels: The x-axis label "Autonomy Support (z-score)" is clear. Still, the y-axis label "Interaction Effect with Diabetes Stigma" could be expanded for clarity (e.g., "Interaction Effect of Autonomy Support on Diabetes Stigma and Self-Management").

Legend: The legend is present but could be more descriptive. For example, "n.s." could be expanded to "Not Significant," and "p < .05" could be labeled as "Significant Interaction Effect."

Range of Observed Data: The range of observed data is marked, but it would be helpful to include a brief explanation of its significance in the figure caption.

Visual Design: The shaded area for "p < .05" could be made more distinct to improve visual clarity.

**Response**: We have made significant changes to figure 3 based on the reviewer’s recommendations.

**Comment 16**: • Highlight the novelty: Emphasize the study's contribution to the limited research on diabetes stigma in low-middle income countries and the role of autonomy support in mitigating its effects.

**Response 17**: We have used the first two sentences of the discussion section to highlight the novelty of the study as suggested.

**Comment 18**: • Compare with global findings: Discuss how the findings align or differ from studies conducted in high-income countries, and what this means for global diabetes care strategies.

**Response**: To the best of our knowledge, this is the only study to assess perceived autonomy support in the context of diabetes stigma. That said, we have attempted to discuss as many related studies as we could find.

**Comment 19**: • Expand on social desirability bias: Discuss how self-reported measures might have influenced the results, particularly in a collectivist society like Ghana, where individuals may feel pressured to provide socially acceptable responses.

**Response**: We have expanded this limitation as suggested.

**Comment 20**: • Cross-sectional design: Highlight the inability to establish causality and suggest future longitudinal studies to confirm the causal relationships between autonomy support, stigma, and diabetes outcomes.

**Response**: We have incorporated this as a limitation.

**Comment 21**: • Sample size and generalizability: Discuss whether the sample size (190 participants) and the recruitment from a single tertiary hospital in Ghana may limit the generalizability of the findings to other populations or settings.

**Response**: We have now highlighted the use of a single site as a limitation for this study.

**Reviewer #3**

**Comment 1**: However, the abstract implies moderation of both behavioral and psychological outcomes, whereas moderation was demonstrated only for diabetes self-management and not for psychological outcomes. This discrepancy should be addressed to ensure consistency between the abstract and the reported results.

**Response**: In the abstract, we report on direct association between autonomy support and all outcomes (including psychological). The moderation/buffering/protective effect of autonomy support is only mentioned in relation to self-management behaviors. We have added the word “directly” to clarify the statement.

**Comment 2**: The authors state that autonomy support has not been investigated in the diabetes-related stigma literature. This claim should be made more cautiously unless supported by a formal scoping or systematic review

**Response**: We have included “to the best of our knowledge” as a caveat to our statement.

**Comment 3**: • The use of purposive and convenience sampling should be justified. While non-probability sampling is common in exploratory and observational research, its rationale should be explained, and implications such as selection bias and limited generalizability should be explicitly discussed.

**Response**: We have mentioned our sampling approach as a limitation.

**Comment 4**: • Details of participant recruitment are insufficient. The manuscript should report how many patients were approached, how many were eligible, how many declined participation, and how many were ultimately included. If available, any systematic differences between participants and non-participants should be noted.

**Response**: We appreciate the reviewer’s comment and agree that detailed recruitment flow chart strengthens transparency. Due to the nature of our recruitment strategy, we are unable to provide number of patients approached, eligible, or declined participation. Specifically, recruitment was conducted through general announcements in the diabetes clinic waiting areas, after which patients who believed they met eligibility criteria voluntarily approached the research team for screening. As a result, we did not systematically track the total number of patients exposed to the recruitment announcement or those who chose not to engage. Additionally, because non-participants were not identified or screened, we are unable to assess systematic differences between participants and non-participants.

**Comment 5**: • The role and training of data collectors are not adequately described.

**Response**: We have now provided additional details regarding the role and training of the research assistants.

**Comment 6**: • Although questionnaires were administered verbally in Twi, details regarding translation procedures (e.g., forward–backward translation, piloting, or cultural adaptation) are missing. The absence of this information represents a significant threat to measurement validity and should be addressed or acknowledged as a limitation.

**Response**: We have noted the lack of appropriate translation and validation studies as limitations

**Comment 7**: • Participant compensation, while ethically approved, should be briefly discussed as a potential source of participation bias.

Response: We do not believe the amount was large enough to cause inducement of participants. We have now clarified this in the method section.

**Comment 8**: • For PROMIS depression and anxiety measures, the manuscript describes conversion from raw scores to T-scores, but it is unclear whether raw scores or T-scores were used in analyses and tables. This should be clearly specified.

**Response**: Thank you. We have now clarified this in the methods section.

**Comment 9**: • There is an inconsistency in the reported duration of diabetes (range 1–55 years in Table 1 versus 1–50 years elsewhere), which should be corrected.

**Response**: We have amended the manuscript to reflect the correct range of 1-55 years

**Comment 10**: • Table numbering and referencing are inconsistent: the text indicates that Table 1 contains bivariate associations, whereas these are presented in Table 2.

**Response**: We have now corrected this error.

**Comment 11:** • The classification of “problematic stigma” as one standard deviation above the mean requires justification through a clear operational definition and rationale, particularly given the absence of a widely accepted clinical cut-off.

**Response**: We have now provided a reference to the original developers of the DSAS-2 scale who suggested this definition for “problematic stigma”
