# Supplementary material for: Investigating the role of perceived autonomy support in moderating the association between diabetes stigma and psychological and diabetes self-management outcomes among adults with type 2 diabetes in Ghana
Source: PLOS Glob Public Health. 2026 Feb 19;6(2):e0006027. doi: 10.1371/journal.pgph.0006027 (PMC12919806; doi:10.1371/journal.pgph.0006027)
Supplement: Attachment — Submitted filename: response_to_reviewers_comments_auresp_2.docx [file pgph.0006027.s006.docx]

Dear Editor,

Thank you for the opportunity to revise our manuscript entitled “Investigating the role of perceived autonomy support in moderating the association between diabetes stigma and psychological and diabetes self-management outcomes among adults with type 2 diabetes in Ghana.” which we submitted to the *PLOS Global Public Health*. We appreciate the time and effort that the editor and reviewers have taken to evaluate our work and provide constructive feedback. We have carefully considered the insightful comments made by the reviewers and have made corresponding amendments to reflect the suggestions made. Changes to the manuscript body are highlighted in yellow and our responses to the reviewers’ comments are summarized below.

**Reviewer #3**

**Comment:** The author has responded to the comment. But a statement on the purpose of selection the sampling strategy can be included.

**Response:** We appreciate the reviewer’s comment. We have highlighted on page 6 line 149 that we chose this strategy to allow us to recruit participants who were readily available at the time of data collection**.**

**Reviewer #2**

**Comment:** Unfortunately, I didn't find the figures (1-3) in the revised file. I couldn't check the revised figures as I suggested more comments in my first review. Therefore, I ask the authors to submit the manuscript once again with the revised files.

**Response**: We appreciate the reviewer’s comments. Figures 1-3 can be found after the “references” section. To view the original file, you may click on the hyperlink at the top right corner of the page where the figure is located. Unfortunately, we are not able to provide the exact page numbers where the figures are located because the online submission system asks us to upload the files separately and later merges them and do not assign page numbers to them.

**Comment**: In revised manuscript, it is very difficult to find out the revision done by the authors. I recommend the authors to provide the response with page number and line number. So that it provides clear idea to reviewer that where the revision took place in the manuscript exactly.

Response: We have reproduced our responses to your earlier comments here but this time, we have included page and line numbers as follows:

**---------------------------- previous comments begin here --------------------------------------**

**Comment 1**: Emphasize that this is one of the first studies to explore autonomy support in the context of diabetes stigma in Ghana, a collectivist society (If so). This would underscore the study's unique contribution.

**Response**: The novelty of the study is now emphasized in the first two sentences of the discussion section. (page 16, line 13-15)

**Comment 2**: The manuscript briefly mentions the hierarchical and collectivist nature of Ghanaian society. Expanding on how this cultural context might influence autonomy support and diabetes stigma could provide a richer understanding of the research.

**Response**: We have now highlighted that the cultural context may influence people to not value autonomy in the same way as individualistic societies might. This has been highlighted in the intro and discussion sections on pages 4 (lines 106-112) and 17 (lines 56-60).

**Comment 3**: “Whereas autonomy support is universal, it may not present in the same manner and may not be as beneficial across cultures (19)”. While the authors mention ross cultures, there should have been many references from different cultures.

**Response**: The cited reference is the source of the idea presented in the statement and not necessarily referencing specific cultures. For clarity, we have now included another reference as suggested. (pages 4-5, lines 112-114)

**Comment 4**: While the manuscript mentions purposive and convenience sampling, it could provide more details about how participants were selected and whether this approach might introduce bias. For example, were there any efforts to ensure diversity in the sample (e.g., age, gender, socioeconomic status)? This has to be stated in the limitation section.

**Response**: We have amended the method section to provide more details about the sampling procedure. We have also noted the use of non-probability sampling as potential source of bias in the limitation section. (page 6, lines 149-157 and page 18, lines 91-92)

**Comment 5**: Meanwhile, I suggest the authors interpret the socioeconomic characteristics of the study population in light of those of studies from other parts of the world with diverse cultures (particularly from high-income countries). This should have been added in the discussion section by using the following suggested articles.

https://doi.org/10.1155/2020/9408316

https://doi.org/10.1016/j.clinthera.2016.07.006

https://doi.org/10.2337/ds23-0013

**Response**: We appreciate the reviewer’s suggestion to discuss socio-economic characteristics of the study population and for providing useful articles for reference. However, we believe that discussing the socioeconomic characteristics of the study sample will distract from the core aims of the manuscript (i.e., perceived autonomy support and stigma). We have another paper under review elsewhere where we do a deep dive on this topic.

**Comment 6**: The manuscript states that surveys were administered via the Qualtrics mobile app. It would be helpful to clarify whether the app was used for both researcher-administered and self-administered surveys, and how data quality was ensured during these processes.

**Response**: We have now clarified that while we offered participants the option to self-administer the survey, none chose this option. So Qualtrics was only used for researcher-administered questionnaire. (page 6, lines 164-165)

**Comment 7**: The statistical methods are well-described, but the rationale for choosing hierarchical multivariable linear regression and the Johnson-Neyman technique could be briefly explained for readers unfamiliar with these methods.

**Response**: We have provided additional rationale to justify the choice of our analytical techniques. (page 9, lines 257-258)

**Comment 8**: While ethical approval and informed consent are mentioned, the manuscript could elaborate on how participant confidentiality and data security were maintained during the study.

**Response**: We have added more information on the steps we took to ensure confidentiality and data security under the section “ethical approval”. (page 9, lines 245-248)

**Comment 9**: “Table 1 shows descriptive statistics.” This should be Table 2.

**Response**: Thank you. We have corrected this error. (page 12 line 292)

**Comment 10**: Table 2: “Perceived autonomy support was negatively and significantly associated with anxiety (r=-0.23) and depressive symptoms (r=-0.27). Participants who reported greater perceived autonomy support tend to engage in better diabetes self-management (r=0.24).” The correlation r<0.3 is usually considered weak. The authors should state this in the text as well.

**Response**: We have rephrased the statements to highlight that the correlation was weak. (page 12 lines 301-304)

**Comment 11**: Table 3: p-value of DSAS-2 in block 2 written as <0.00001, and this should be corrected as <0.0001. Please check that all the decimals are consistent.

**Response**: Thank you. We have corrected this error. (page 15)

**Comment 12**: Figures: Ensure figures are high-resolution for better readability, especially for publication. Use consistent font sizes, styles, and colors across all figures for a professional appearance. The captions should provide a brief explanation of the figure's content, including the key findings and any critical annotations.

**Response**: We have now included higher resolution figures in the manuscript and have added more details to the captions (all figures are located after “references”; no page or line number assigned)

**Comment 13**: Figure 1

Clarity of Labels: The axis labels are not fully descriptive. For example, "Diabetes-related stigma" could be labeled as "Diabetes Stigma (DSAS-2 Score)" for clarity. Similarly, "Anxiety symptoms" and "Depressive symptoms" could be labeled with their respective scales (e.g., "Anxiety Symptoms (PROMIS Anxiety T-Score)" and "Depressive Symptoms (PROMIS Depression T-Score)").

Data Points: The scatter plot appears cluttered, making it difficult to interpret the relationships. Consider reducing the size of the data points or using transparency to make overlapping points more distinguishable.

Trend Lines: The trend lines are present, but their labels or legends are missing. Adding a legend or annotations to clarify the meaning of the trend lines would improve readability.

**Response**: We appreciate this detailed feedback. We have amended figure 1 to reflect your suggestions. We have clarified the meaning of the trend lines in the figure’s caption. (all figures are located after “references”; no page or line number assigned)

**Comment 14**: Figure 2

Axis Labels: The x-axis label "T2D stigma z-scores" could be expanded to "Diabetes Stigma (Standardized Z-Scores)" for clarity. The y-axis label "Autonomy support" is unclear. It could be updated to "Interaction Effect with Diabetes Stigma" to reflect better the data being presented.

Legend: The figure lacks a legend explaining the meaning of the different lines (e.g., "Mean z-score," "1 SD," etc.). Adding a legend would make the figure more straightforward to interpret.

Visual Design: The figure could benefit from a more apparent distinction between the shaded areas (e.g., using different colors or patterns for "n.s." and "p < .05"). The dashed vertical line could be labeled to indicate its significance (e.g., "Threshold for significant moderation effect").

**Response**: We thank the reviewer for these useful observations. Please note that figure 2 is a standard “spotlight” interaction plot. We have added additional details in the caption to clarify the plot’s interpretation. We have also modified the axis labels. (all figures are located after “references”; no page or line number assigned)

**Comment 15**: Figure 3

Axis Labels: The x-axis label "Autonomy Support (z-score)" is clear. Still, the y-axis label "Interaction Effect with Diabetes Stigma" could be expanded for clarity (e.g., "Interaction Effect of Autonomy Support on Diabetes Stigma and Self-Management").

Legend: The legend is present but could be more descriptive. For example, "n.s." could be expanded to "Not Significant," and "p < .05" could be labeled as "Significant Interaction Effect."

Range of Observed Data: The range of observed data is marked, but it would be helpful to include a brief explanation of its significance in the figure caption.

Visual Design: The shaded area for "p < .05" could be made more distinct to improve visual clarity.

**Response**: We have made significant changes to figure 3 based on the reviewer’s recommendations. (all figures are located after “references”; no page or line number assigned)

**Comment 16**: • Highlight the novelty: Emphasize the study's contribution to the limited research on diabetes stigma in low-middle income countries and the role of autonomy support in mitigating its effects.

**Response 17**: We have used the first two sentences of the discussion section to highlight the novelty of the study as suggested. (page 16 lines 13-15)

**Comment 18**: • Compare with global findings: Discuss how the findings align or differ from studies conducted in high-income countries, and what this means for global diabetes care strategies.

**Response**: To the best of our knowledge, this is the only study to assess perceived autonomy support in the context of diabetes stigma. That said, we have attempted to discuss as many related studies as we could find. (pages 16-18)

**Comment 19**: • Expand on social desirability bias: Discuss how self-reported measures might have influenced the results, particularly in a collectivist society like Ghana, where individuals may feel pressured to provide socially acceptable responses.

**Response**: We have expanded this limitation as suggested. (page 18, lines 85-89)

**Comment 20**: • Cross-sectional design: Highlight the inability to establish causality and suggest future longitudinal studies to confirm the causal relationships between autonomy support, stigma, and diabetes outcomes.

**Response**: We have incorporated this as a limitation. (page 18, lines 85-92)

**Comment 21**: • Sample size and generalizability: Discuss whether the sample size (190 participants) and the recruitment from a single tertiary hospital in Ghana may limit the generalizability of the findings to other populations or settings.

**Response**: We have now highlighted the use of a single site as a limitation for this study. (page 18, lines 85-92)
